# Centrifugal Inertia‐Induced Directional Alignment of AgNW Network for Preparing Transparent Electromagnetic Interference Shielding Films with Joule Heating Ability

**DOI:** 10.1002/advs.202406758

**Published:** 2024-08-08

**Authors:** Weijun Zhao, Jingwen Dong, Zhaoyang Li, Bing Zhou, Chuntai Liu, Yuezhan Feng

**Affiliations:** ^1^ State Key Laboratory of Structural Analysis Optimization and CAE Software for Industrial Equipment National Engineering Research Center for Advanced Polymer Processing Technology Zhengzhou University Zhengzhou 450002 China

**Keywords:** AgNW network, directional alignment, electromagnetic interference shielding, Joule heating, transparent conductive film

## Abstract

Transparent electromagnetic interference (EMI) shielding is highly desired in specific visual scenes, but the challenge remains in balancing their EMI shielding effectiveness (SE) and optical transmittance. Herein, this study proposed a directionally aligned silver nanowire (AgNW) network construction strategy to address the requirement of high EMI SE and satisfactory light transmittance using a rotation spraying technique. The orientation distribution of AgNW is induced by centrifugal inertia force generated by a high‐speed rotating roller, which overcomes the issue of high contact resistance in random networks and achieves high conductivity even at low AgNW network density. Thus, the obtained transparent conductive film achieved a high light transmittance of 72.9% combined with a low sheet resistance of 4.5 Ω sq^−1^ and a desirable EMI SE value of 35.2 dB at X band, 38.9 dB in the K‐band, with the highest SE of 43.4 dB at 20.4 GHz. Simultaneously, the excellent conductivity endowed the film with outstanding Joule heating performance and defogging/deicing ability, ensuring the visual transparency of windows when shielding electromagnetic waves. Hence, this research presents a highly effective strategy for constructing an aligned AgNW network, offering a promising solution for enhancing the performance of optical‐electronic devices.

## Graphical abstract

A novel rotation spraying technique with centrifugal inertia inducing effect is used to construct an oriented alignment AgNW network, that reveals a 72.9% transmittance, a 4.5 Ω sq^−1^ sheet resistance, and a desirable EMI shielding with the highest SE of 43.4 dB at 20.4 GHz, simultaneously shows an outstanding Joule heating and defogging ability to ensure the visual transparency when shielding electromagnetic waves.

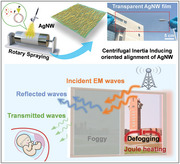

## 1 Introduction

The rapid advancement of wireless communication technology brings convenience to individuals and generates serious electromagnetic interference (EMI) pollution, reducing the accuracy and reliability of electronic equipment during operation and seriously threatening people's health.^[^ ^1^, ^2^, ^3^, ^4^, ^5^, ^6^ ^]^ The development of high‐performance EMI shielding material is of utmost urgency. Specifically, flexible EMI shielding materials with high transmittance are highly desired in many visual scenes, such as space detection^[^ ^7^ ^]^ and receiving optical windows,^[^ ^8^ ^]^ precision instrument displays,^[^ ^9^ ^]^ and medical electromagnetic isolation observation windows.^[^ ^10^ ^]^ Excellent EMI shielding efficiency (EMI SE) requires outstanding electrical conductivity; however, excellent conductivity is inseparable from the dense conductive network structure, which will inevitably reduce the light transmittance. Therefore, identifying how to uniquely balance electrical conductivity, EMI shielding performance, and visible light transmittance of transparent conductive films is the key to promoting the rapid development of optoelectronic devices.^[^ ^11^ ^]^

1D silver nanowire (AgNW) with a high aspect ratio and excellent electrical conductivity exhibits an intensive potential in the field of transparent EMI shielding and is considered the most promising material to replace indium tin oxide (ITO).^[^ ^12^, ^13^ ^]^ For example, Zhu et al.^[^ ^14^ ^]^ reported a transparent EMI shielding film based on a random AgNW network using a simple Mayer‐rod coating method, which exhibited a sheet resistance of 22 Ω sq^−1^ with a transmittance of 95.5%. Despite the encouraging advances, however, the persistence issue with random networks is the presence of abundant junctions that not only increase the contact resistance of the network but also decrease the transmittance by reducing the void ratio. The rational design of the AgNW network structure is an efficient strategy to significantly enhance the electrical conductivity and optical transmittance simultaneously, including prefabricated mesh and orientation alignment.^[^ ^15^, ^16^ ^]^ The prefabricated mesh structure can regulate the distribution of AgNW to form a regular conductive network. Moreover, the grid gaps ensure sufficient optical transmittance, producing high optical‐electrical and EMI shielding properties. Unfortunately, prefabricated mesh structures have issues, such as Moiré phenomena, pattern visibility, and lack of flexibility.^[^ ^17^ ^]^ Conversely, the oriented alignment conductive network maximizes the use of 1D AgNWs by reducing ineffective junctions, thus reducing the sheet resistance and avoiding the occurrence of local short circuits and leakage. Combined with the similar light transmittance with a random network, the orientation conduction network thus shows better optical‐electrical performance.

To date, various techniques have been developed to align AgNW in an ordered manner, such as shear coating,^[^ ^18^ ^]^ brush coating,^[^ ^19^ ^]^ strain‐release assembly,^[^ ^20^ ^]^ microfluidic alignment,^[^ ^21^ ^]^ and evaporation‐induced assembly.^[^ ^22^ ^]^ For example, Chen et al.^[^ ^23^ ^]^ utilized superwetting‐induced technology to transfer the self‐assembled complex consisting of AgNWs and ionic liquid to obtain an ordered AgNW structure, resulting in transparent electrodes with excellent optoelectronic properties of ≈88% transmittance and sheet resistance of 46 Ω sq^−1^. Hu et al.^[^ ^16^ ^]^ reported a method of self‐assembly using fluid agitation to achieve the orientation distribution of AgNW. They used cross‐assembly to obtain a transparent conducting AgNW network with up to 85% transparency and a surface resistance of only 2.8 Ω sq^−1^. Very recently, our group developed highly oriented AgNW‐based transparent shielding films using a pre‐stretching method,^[^ ^12^ ^]^ where the high connectivity of the fence‐like AgNW network endows the final film with excellent photoelectric performance (7.68 Ω sq^−1^ at 73.7% transmittance) and satisfactory EMI shielding performance (32.2 dB). Nevertheless, these techniques require complicated processes or specialized equipment such as additional transfer processes, pre‐grown vertical AgNW arrays, and special substrates, which restrict the cost‐effective and scalable assembly of highly aligned AgNW in a controlled manner.

In this research, inspired by the high‐speed rotation receiving device in electrospinning, we demonstrated a facile strategy for preparing a highly transparent EMI shielding film composed of directionally aligned AgNW generated using a rotation spraying technique. In this process, the centrifugal inertia force induced by the high‐speed rotating roller can drive the AgNW with a high aspect ratio to align in the rotation direction. Compared with traditional spraying, the centrifugal inertia force generated by this approach can remove excess solvent and aggregate of conductive fillers, achieving the perfect combination of high light transmittance, high EMI SE, and high Joule heating capability. Typically, the alignment of the AgNW network significantly enhances the aligned AgNW film to simultaneously exhibit a low sheet resistance of 4.5 Ω sq^−1^, a light transmittance of 72.9%, and an outstanding EMI SE of 35.2 dB. Furthermore, the excellent conductivity of the transparent shielding film allows the generation of satisfactory Joule heating (102 °C @ 4 V), ensuring its application in extreme conditions. These outstanding attributes of aligned AgNW films make them suitable for various applications in visual windows, aerospace equipment, and medical health.

## 2 Results and Discussion

### 2.1 Fabrication of Directionally Aligned AgNW Networks

**Figure 1:**
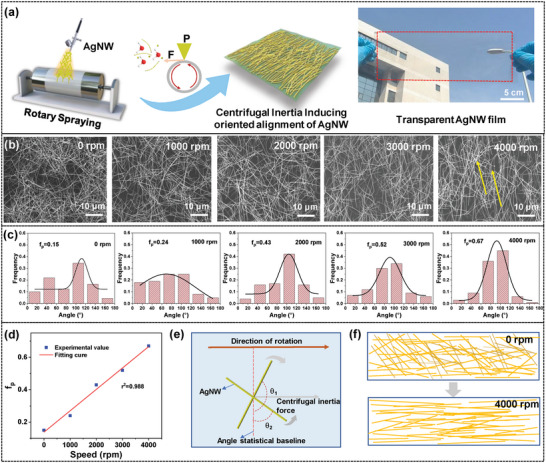
a) Illustration of rotation spraying for fabricating directionally aligned AgNW network. b) SEM images and c) statistical diagram of AgNW angels in networks with different rotation speeds. d) AgNW orientation factors (*f_p_*) as the function of rotation speed. e) Statistical diagram of the angle θ between AgNW and the vertical direction of roller rotation. f) AgNW alignment mechanism diagram.

**Figure** **1**a illustrates the preparation process of transparent EMI shielding film with directionally aligned AgNW networks using a novel rotation spraying method. In brief, a hydrophilic polycarbonate (PC) film treated with oxygen plasma is chosen as the substrate for good adhesion to the AgNW coating. AgNW with a high aspect ratio of >300 synthesized by the polyol reduction method (Figure S1, Supporting Information) is sprayed on the surface of a high‐speed rotating PC substrate, achieving the directional alignment of AgNW along the direction of rotation, and obtaining a large‐area transparent conductive film with high optical transparency. In the spraying process, the flat receiving device of traditional spraying technology is upgraded to a high‐speed rotating roller (Figure S2, Supporting Information). Compared with the traditional spraying process, the high‐speed rotating roller generates a centrifugal inertia force, which can remove excess solvent and the aggregate of conductive fillers, enhancing the uniformity of AgNW coating. Thus, the centrifugal inertia force can effectively drive the directional alignment of 1D AgNW and form an aligned AgNW network after solvent evaporation.

The orientation of AgNW is expected to improve by increasing the centrifugal inertial force. During the alignment process, the centrifugal inertial force can be calculated as F = mω^2^ *r* = *m*(π*n*/30)^2^ *r*, where *m* is the mass of an ideal single AgNW (4.64 × 10^−12^ g, Figure S3, Supporting Information), *n* is the rotation speed of the roller (1000, 2000, 3000, and 4000 rpm), and *r* is the radius of the roller (4.5 cm). The result indicates that the centrifugal inertial force acting on the single AgNW increases gradually as the roller speed is enhanced (Table S1, Supporting Information). Thus, the directionally aligned AgNW network can be effectively controlled by adjusting the roller speed. For this reason, the significance of the roller speed on the orientation of AgNW network is emphatically investigated. Figure 1b demonstrates that without rotation speed (0 rpm), a randomly distributed AgNW network is formed on the PC substrate after spraying. Interestingly, introducing a high‐speed rotating substrate into the traditional spraying process can form a more orderly AgNW network along the roller rotation direction. In addition, too high rotational speed may damage the AgNW network, because too high centrifugal force may cause some of the weak bonding silver nanowires to separate from the substrate surface. So, the highest rotation speed was set as 4000 rpm in this experiment. Generally, the orientation index (*f_p_*) of the AgNW network can be achieved by an exponential formula proposed by Fakirov and Fakirova as follows:^[^ ^24^ ^]^

(1) $$f_{p}=2\langle cos^{2}\theta \rangle -1$$

(2) $$\langle cos^{2}\theta \rangle =\frac{\sum N\left(\theta \right)cos^{2}\theta }{\sum N\left(\theta \right)}$$

Where N (θ) represents the occurrence frequency of angle θ between AgNW and the vertical direction of roller rotation (Figure 1e). Specifically, *f_p_* = 0 manifests that the arrangement is chaotic, whereas  *f_p_* = ±1 refers to a completely oriented arrangement in parallel rotation directions (θ = 0° or 180°). Figure 1c depicts the statistical distributions of the angle θ for aligned AgNW networks obtained under different rotation speeds. The network without rotation has an asymmetric distribution of angle θ between 0° and 180°, with an *f*_p_ value of merely 0.15, suggesting that the AgNW network is randomly distributed. Conversely, the angle θ of the films on a high‐speed rotating substrate is focused in a symmetrical distribution centered ≈90°. As the rotation speed increases from 1000 to 4000 rpm, the angle θ becomes more concentrated at 90°, and the corresponding *f_p_* increases from 0.24 to 0.67, with a good linear relationship between *f_p_* value and rotation speed (Figure 1d). This result confirms that the increase in rotation speed enhances the orientation of AgNW alignment, implying that the orientation alignment of AgNW is linearly controllable by adjusting the rotation speed.

Furthermore, the alignment of AgNW is also influenced by the interfacial interaction, which determines the rotational resistance between AgNW and substrate. The spectra obtained from a Fourier transform infrared spectrometer (FTIR) were used to confirm the interaction between PC and AgNW coated with Poly(vinylpyrrolidone) (PVP), where the C ═ O peak of PC shifts toward a lower wavenumber relative to PC/AgNW film (Figure S4, Supporting Information), indicating the presence of weak hydrogen bonding between the units. Therefore, the weak interfacial interaction implies the low rotational resistance between AgNW and substrate, which allows AgNWs to rearrange in the direction of rotation due to the effect of centrifugation inertia force (Figure 1e), forming a directionally aligned AgNW network (Figure 1f). Based on the above analysis, it can be inferred that directionally aligned AgNW networks can be easily controlled using our upgraded rotation spraying method. It is significant for optimizing optical‐electrical performance and improving EMI shielding capability.

### 2.2 Optical and Electrical Properties

**Figure 2:**
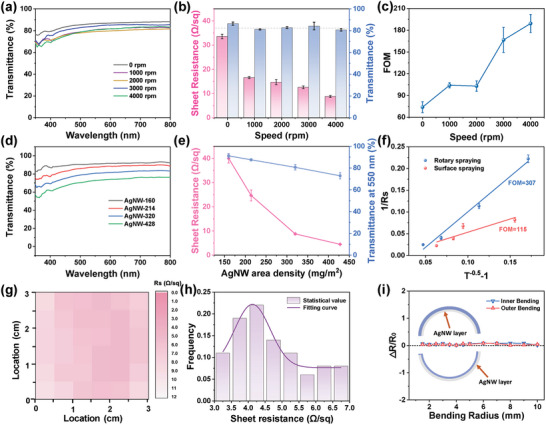
a) Optical transmittance, b) sheet resistance and light transmittance (550 nm), and c) FOM value of PC/AgNW film with different rotation speed. d) Optical transmittance and e) sheet resistance and light transmittance (550 nm) of aligned AgNW network with various AgNW coating density. f) Experimental data and linear fitting of 1/Rs versus T^−0.5^‐1. g) Rs distribution and h) corresponding resistance statistics histogram. i) ΔR/R_0_ as a function of bending radius (3 mm) (ΔR and R_0_ represent the resistance change and the initial resistance, respectively).

**Figure** **2**a,b compare the optical‐electrical properties of AgNW networks (320 mg m^−2^) obtained at various rotation speeds. Increasing rotation speed from 0 to 4000 rpm decreases sheet resistance, *R_s_* value from 33.6 to 8.8 Ω sq^−1^, and the corresponding light transmittance remains almost constant (≈80%). This is mainly because the directional alignment of AgNW in the network can reduce the ineffective junctions compared with the random network, thus constructing more effective conductive pathways and reducing *R_s_* at the same coating density of AgNW. To better evaluate the comprehensive optical‐electrical performance of the aligned AgNW network, a figure of merit (FoM) is introduced, which is defined as the ratio of electrical conductance (σ_dc_) to optical conductance (σ_op_) and calculated using the following formula:^[^ ^25^, ^26^ ^]^

(3) $$\mathrm{FoM}\;=\sigma_{dc}\;/\;\sigma_{op}=Z_{0}/\left(2R_{s}\cdot \left(T^{-1/2}-1\right)\right)$$

Where Z_o_ and *T* refer to the impedance of free space (377 Ω) and light transmittance (λ = 550 nm), respectively. As shown in Figure 2c, the gradually enhanced FoM values with the increase of rotation speed confirm the improvement of the comprehensive optical‐electrical performance by a directionally aligned AgNW network. Generally speaking, the higher the rotation speed, the better the directional alignment, corresponding to the more excellent optical‐electrical performance.

By changing the coating density of AgNW, the transparent conductive film can be optimized to achieve satisfactory optical‐electrical performance.^[^ ^27^ ^]^ Figure 2d,e show that the aligned AgNW films exhibit satisfactory optical transmittance (91.2−72.9% at 550 nm) and low *R_s_* (40–4.5 Ω sq^−1^) as a function of the coating density of AgNW. In other words, high conductivity can be achieved by constructing denser conductive networks with increased coating density of AgNW (Figure S5, Supporting Information), but the light transmittance will inevitably be reduced. To demonstrate the advantage of optical‐electrical performance compared with the random network (Figure S6, Supporting Information), the FoM derived from Equation (3) is used. As shown in Figure 2f, the higher FoM value of the aligned AgNW film (FoM = 307) than that of random AgNW film (FoM = 115) confirms the better optical‐electrical performance for the directional aligned AgNW network, which proves the advantage of our rotation spraying in preparing high‐performance AgNW‐based transparent film comparing to conventional spraying.

The uniform spatial distribution of the AgNW network is also a crucial parameter for evaluating the optical‐electrical performance.^[^ ^21^, ^28^ ^]^ For this reason, the *R_s_* value of each pixel in a 0.5 × 0.5 array of aligned AgNW‐320 film (3 × 3 cm^2^) is investigated. As shown in Figure 2g, the *R_s_* distribution in the whole region shows good uniformity with a small standard deviation of 1.19 (Figure 2h), indicating that the aligned AgNWs on the substrate are uniformly distributed. Similarly, the uniformly distributed AgNW network can be demonstrated by the uniform temperature distribution for the film subjected to a 2 V voltage with a standard deviation of 1.15 (Figure S7, Supporting Information). Moreover, the flexibility of aligned AgNW film is demonstrated by testing the relative resistance variation (△R/R_0_) at different bending radii. As depicted in Figure 2i, regardless of whether the sample is bent inward or outward, even with a bending radius of 1 mm, its Δ R/R_0_ value remains consistently stable at ≈0. The aligned AgNW film also exhibits durable flexibility that can withstand 1000 bending cycles (inter or outer bending) without obvious change in sheet resistance (Figure S8, Supporting Information).

### 2.3 EMI Shielding Performance

**Figure 3:**
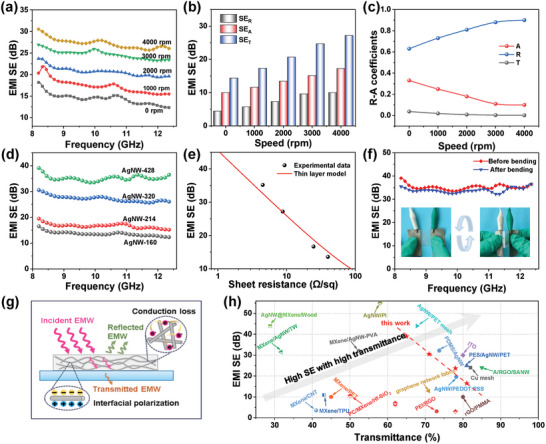
a) EMI shielding performance in X‐band, b) corresponding average SE_T_, SE_A_, and SE_R_, and c) average power coefficient of AgNW film with different rotation speeds. d) Total EMI shielding performance in X‐band of aligned AgNW film with different coating density. e) Experimental data and linear fitting of EMI SE versus R_s_. f) Comparison of EMI SE before and after 1000 bending cycles (r = 3 mm). g) EMI shielding mechanism. h) Comparison of EMI SE versus light transmittance of aligned AgNW film with other transparent EMI shielding films.

The low sheet resistance of AgNW film results in high impedance mismatching with space, thus inducing abundant reflection when electromagnetic waves radiate.^[^ ^29^, ^30^ ^]^ **Figure** **3**a reveals the EMI shielding performance of AgNW films with different rotation speeds in the X‐band. With the rotation speed increasing from 0 to 4000 rpm, the EMI SE exhibits a significant upward trend, with the highest SE of 30.5 dB at 8.2 GHz for the 4000 rpm film, which meets the requirement of commercial application.^[^ ^31^ ^]^ According to Schelkunoff's functions,^[^ ^32^ ^]^ the increased conductivity for the aligned AgNW networks induced by the increase of rotation speeds can be attributed to the improvement in impedance mismatching and conduction loss, thus resulting in the gradual increase in the SE_R_ and SE_A_ (Figure 3b). Furthermore, the dominant EMI shielding mechanism is judged by reflection (R) and absorption (A) coefficients. As shown in Figure 3c, the random AgNW film showing similar R and A values reveals that reflection and absorption are equally important in its shielding mechanism. The increase in rotation speed leads to an increase in conductivity, which in turn causes a gradual increase in the R‐value; however, it also decreases the A value. The aligned film prepared at 4000 rpm exhibits the highest R‐value of 0.9 and the lowest A‐value of 0.1. This implies that the shielding mechanism transforms from absorption/reflection coexistence for random AgNW films to reflection domination for aligned AgNW films.^[^ ^33^, ^34^, ^35^ ^]^

Increasing the coating density of AgNW can significantly enhance the conduction network density. It can effectively enhance the EMI shielding performance of aligned AgNW films both in the X‐band and K‐band (Figure 3d; Figure S9, Supporting Information), showing the maximum average EMI SE value of 35.2 dB in the X‐band and 38.9 dB in the K‐band with ≈72.9% transmittance. Especially, the highest EMI SE reached a value of 43.4 dB at a frequency of 20.4 GHz, indicating that more than 99.99% of EM waves were shielded as they passed through the aligned AgNW film. Moreover, as the coating density of AgNW increases, the values of SE_R_ and SE_A_ in the X‐band and K‐band also increase gradually, attributing to the improvement of electrical conductivity (Figure S10a,c, Supporting Information). According to the transmission line theory,^[^ ^36^, ^37^ ^]^ the impedance mismatch induced by the enhancement of electrical conductivity leads to an increase in SE_R_ value, while the increase in ohmic losses caused by the dense AgNW network enhances the SE_A_ value.^[^ ^29^ ^]^ Furthermore, the gradually increased R‐value is much higher than the corresponding A‐value in Figure S10b, d, reinforcing the reflection domination mechanism with the increasing coating density of AgNW. Especially in the K‐band, the highest R‐value is greater than 0.97, which means that 98.5% of the incident EM waves are reflected on the surface of the shielding film before being incident on the shielding film. Moreover, a thin layer model (SE  =  20log(1 +Z_0_/2R_s_), where Z_0_ of 377 Ω represents the wave impedance in free space) is used to accurately estimate the EMI shielding performance.^[^ ^12^ ^]^ As shown in Figure 3e, the correlation between *R_s_* and EMI SE obtained by the model equation is consistent with the experimental data, indicating that the EMI SE values of the aligned AgNW film can be effectively evaluated based on the *R_s_* of the sample. Besides, because of the high flexibility, the aligned AgNW film demonstrates the EMI shielding stability without obvious performance degradation when suffering a long‐term cycled bending (Figure 3f), which is crucial for the practical application of wearable electronics.

Figure 3g depicts the transmission state of EMI waves in aligned AgNW film, which more intuitively explains its EMI shielding mechanism. Essentially, there is a dominated EMI shielding mechanism by reflection loss when the incident EM waves reach the conductive layer of the transparent shielding film, impedance mismatch is caused by the high‐density free electrons in the excellent conductive network of AgNWs, leading to most of the EM waves are rapidly reflected.^[^ ^38^ ^]^ Then, the incident EM waves generate ample ohmic loss when interacting with the AgNW conductive layer with electron carriers, resulting in vast attenuation of EM waves. At the same time, EM waves are also weakened due to differences in polarity or conductivity between the AgNW conductive layer and substrate, which result in the transmittance of a very small amount of EM wave while showing efficient EMI shielding performance.^[^ ^39^, ^40^ ^]^ Moreover, compared with the previously reported transparent EMI shielding film (Figure 3h), our aligned AgNW films exhibit significant advantages in balancing EMI SE and optical transmittance. Specifically, the EMI SE is superior to graphene‐based,^[^ ^41^ ^]^ MXene‐based,^[^ ^42^ ^]^ and most AgNW‐based films^[^ ^28^ ^]^ at the same light transmittance.

### 2.4 Joule Heating Performance

**Figure 4:**
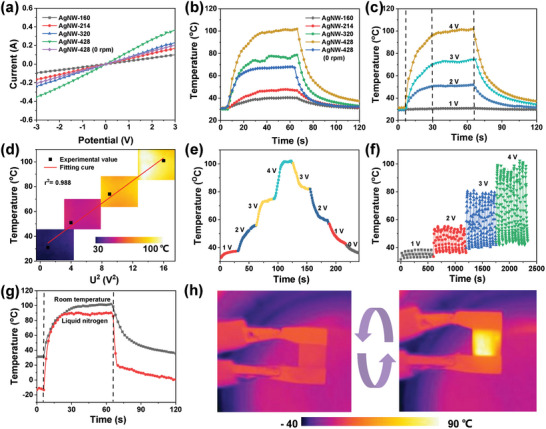
a) *I–V* curves and b) temperature‐time curves of aligned AgNW film with various AgNW densities and random AgNW film (AgNW‐428 (0 rpm)). c) Electrothermal conversion curves of AgNW‐428 film at different input voltages. d) The saturation temperature as a function of U^2^. e) Temperature change curves of AgNW‐428 film under stepwise‐increased/decreased voltages. f) Cyclic stability of AgNW‐428 film at various applied voltage. g) Real‐time surface temperature of AgNW‐428 film in different environments and h) corresponding infrared thermal images.

The exceptional electrical conductivity of the aligned AgNW film also endows its excellent Joule heating performance, ensuring its stable and visual EMI shielding performance under low temperature or foggy environments.^[^ ^43^ ^]^ **Figure** **4**a depicts the typical linear voltage‐current characteristic of AgNW films with different area densities, where all curves pass through the origin, which implies an excellent Joule heating effect and is consistent with conductivity results. The Joule heating effect can be determined using Joule's law: $Q\;=\frac{U^{2}}{R}\;t$, where Q, U, R, and t represent the generated heat, input voltage, resistance, and heating time, respectively.^[^ ^44^, ^45^ ^]^ As shown in Figure 4b, all samples reach the saturation temperature within 20 s when applying a voltage of 4 V and gradually decrease to room temperature once the power supply is disconnected. With the increase in coating density of AgNW, the saturation temperature is prominently enhanced with the maximum value greater than 100 °C for AgNW‐428 film due to its highest conductivity. Meanwhile, with the same area density, due to the difference in conductivity, the saturation temperature of oriented film at steady state is much higher than that of non‐directional components. Figure 4c depicts the electrical heating behavior of AgNW‐428 film with increasing applied voltage from 1 to 4 V, and the corresponding saturation temperature rises from 35 to 102 °C, which is much higher than the random network. (Figure S11, Supporting Information). According to Joule's law, the equation $Q\;=\frac{U^{2}}{R}\;t$ shows that the saturation temperature is well linear with the square of the input voltage (Figure 4d). Besides, the corresponding thermal infrared images with uniform temperature distribution indicate satisfactory Joule heating homogeneity for aligned AgNW film due to its uniform sheet resistance (Figure 4d, inset).

The controllability and stability of the Joule heating function are essential in thermal management applications.^[^ ^46^ ^]^ For our AgNW‐428 film, the surface temperature can be quickly and smoothly customized by adjusting the applied voltage from 0–4 V or 4–0 V, which reveals the highly sensitive and controllable Joule heating ability (Figure 4e). The stability and reliability of Joule heating can be demonstrated by the cycle heating test (Figure 4f), wherein the temperature curve associated with the loading and unloading of input voltage presents a stable and regular rise and fall. Moreover, the Joule heating testing was carried out in a liquid nitrogen vapor environment to explore the possibility of the aligned AgNW film as an electric heater in an extremely cold environment. As shown in Figure 4g, the film can quickly reach saturation temperature, with a slight decrease (5 °C) compared to the test at room temperature. Moreover, the film maintains relatively stable and cyclic surface heating in the liquid nitrogen environment (Figure 4h). This controllable, stable, and frigostable Joule heating ability enhances the potential applications in different scenarios.

### 2.5 EMI Shielding Application

**Figure 5:**
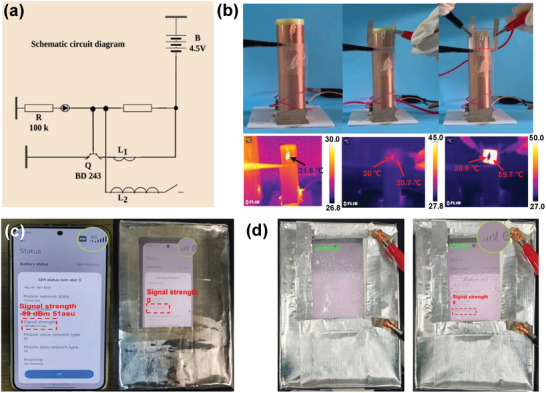
a) Circuit diagram of the Tesla coil. b) Images of diode changes without or with PC/AgNW film under the action of Tesla coil and effect of PC/AgNW film at a constant voltage of 2 V on diode variation. c) Images of smartphone signal changes without or with PC/AgNW film. d) Shielding effect of PC/AgNW film without or with Joule heating deicing on smartphone signal.

We designed a wireless power transmission system based on the Tesla coil shown in **Figures** **5**a and S12 (Supporting Information) to visualize the EMI shielding ability in different scenarios. When the circuit is switched on, the changing current in the primary coil induces a high voltage in the secondary coil. It forms an electromagnetic field, which causes the LED to light up under a small current generated by electromagnetic induction (Figure 5b). When the aligned AgNW film is placed between the coil and the LED, the LED turns off, confirming that the film can block the transmission of EM waves. Interestingly, when a voltage (2 V) is applied to this film, the corresponding infrared thermal image confirms the Joule heating function while maintaining the function of shielding electromagnetic signals (i.e., the LED remains extinguished).

One important function of Joule heating is the defogging or deicing ability, which is expected to maintain high optical transparency for visual EMI shielding in foggy or frosty conditions.^[^ ^47^ ^]^ Figure 5c shows that a smartphone placed in an open aluminum foil box can still be used normally, with a signal strength of ‐89 dBm 51asu. After the aluminum foil box window is covered with the aligned AgNW film, the smartphone signal is completely shielded with zero signal strength. Furthermore, in a cold and humid environment, the shielding window covered with frost loses the visualization function with low transparency (Figure 5d). When both the shielding window is applied by a power supply with a voltage of 10 V, the frost on its surface quickly disappears due to the Joule heating effect, and the window again presents a visual state with the no‐signal state of mobile being visually sighted. The combination of Joule heating and EMI shielding implies that the aligned AgNW film has good potential in smart electromagnetic protection windows.^[^ ^33^ ^]^

## 3 Conclusion

In summary, a novel rotation spraying method was used to fabricate the transparent conductive film with a directionally aligned AgNW network. The film demonstrated comprehensive properties, including satisfactory light transmittance, excellent EMI shielding performance, and Joule heating performance. The orientation distribution of the AgNW network is usually induced by the centrifugal inertia force generated by the high‐speed rotation roller, leading to the best optical‐electric performance, characterized by a transmittance of 72.9% and *R_s_* of 4.5 Ω sq^−1^. The aligned AgNW film can achieve a satisfactory EMI SE of 35.2 dB in the X‐band. Furthermore, the remarkable flexibility of this film can maintain a stable EMI shielding performance after 1000 bending cycles. Furthermore, the transparent conductive film exhibits excellent Joule heating performance (102 °C at 4 V), which can be used to ensure the stable and visual EMI shielding function under low temperatures or foggy environments. The directional alignment structure can overcome the trade‐off between the electrical conductivity and optical transmittance in random AgNW networks, making it one of the best AgNW‐based transparent EMI shielding film technologies.

## 4 Experimental Section

### Materials

Poly(vinylpyrrolidone) (PVP, K30: M_w_ = 55 000 g mol^−1^ and K90: M_w_ = 360 000 g mol^−1^) was obtained from Aladdin Reagent Co., Ltd. Silver nitrate (AgNO_3_, AR), Ferric chloride (FeCl_3_, AR), ethanol glycol (EG, AR), and ethanol were procured from Sinopharm Chemical Reagent Co., Ltd. Polycarbonate (PC) film with optical transmittance of 90.5% was purchased from General Electric. All chemicals were used without any further purification.

### Synthesis of AgNW

As noted in previous studies,^[^ ^28^ ^]^ AgNW was synthesized using a modified polyol method. First, 0.05 g of PVP (K30) and 0.10 g of PVP (K90) were added to 22 mL of EG with strong stirring at room temperature to obtain a uniform mixture solution. Then, the prefabricated FeCl_3_ solution (2.5 mL, 0.6 m in EG) was slowly added to the above solution under continuous agitation. After the reaction solution was heated to 140 °C, the prefabricated AgNO_3_ solution (3 mL, 60 mg mL^−1^ in EG) was slowly added into it drop by drop. Once the reaction solution turns gray, immediately stop stirring and continue heating at 140 °C for 60 min. Subsequently, a substantial quantity of ethanol was added to stop the reaction, and the resulting substances were washed by centrifugation with deionized water (5000 rpm, 10 min) multiple times to remove impurities. Finally, a high‐quality AgNW aqueous dispersion (1.5 mg mL^−1^) was obtained.

### Preparation of AgNW‐Aligned Transparent Conductive Film

As shown Figure 1a, PC film was used as a coating substrate, pretreated with oxygen plasma, and attached to the high‐speed rotating drum for spraying. To explore the effect of rotation speed on the orientation arrangement of AgNW, a fixed amount of AgNW solution (3 mL) was uniformly sprayed on the high‐speed rotating substrate, where the speed was set at 1000, 2000, 3000, and 4000 rpm. Furthermore, to investigate the effect of AgNW network density on photoelectric performance, AgNW solution with different volumes (1.5, 2, 3, and 4 mL) was sprayed on the substrate at 4000 rpm by a commercial Airbrush (Pistole AFC‐101A) with a spraying pressure of 0.5 MPa and a distance of 5 cm. The obtained transparent conductive films were named AgNW‐*x*, where *x* is the loading density of AgNW. The loading density of AgNW is calculated using the following equation:

(4) $$\sigma_{A}\left(mg/m^{2}\right)=\left(V_{A}\times c_{A}\right)/S_{PC}$$

Where σ_*A*_, *V_A_*, *c_A_*, and *S_PC_* are the loading density of AgNW, the spraying volume, the mass fraction of AgNW solution, and the spraying area of the PC substrate, respectively. The loading densities of AgNWs were determined to be 160, 214, 320, and 428 mg m^−2^, corresponding to AgNW‐160, AgNW‐214, AgNW‐320, and AgNW‐428, respectively. In contrast, random AgNW networks (0 rpm) were prepared by traditional spraying process under the same condition.

### Characterizations

Morphology was characterized using a scanning electron microscope (SEM, Zeiss MERLIN Compact). Structure and chemistry were characterized using a Fourier transform infrared spectrometer (FTIR, Nicolet 6700). Current–voltage (*I–V*) characterization was accomplished using an RST 5000‐type electrochemical workstation. Thermogravimetric analysis (TGA) was recorded using a Netzsch TG209F3 thermal instrument. Optical transmittance was measured using an UV–vis spectrophotometer (PerkinElmer Lambda 1050+, USA), and the baseline on the UV–vis test was corrected using a PC substrate. Sheet resistance was measured using a four‐point probe instrument (RST‐8, Guangzhou Four Point Probe Technology), and the resistance change under different bending conditions was recorded using the Tektronix DMM 4050 multimeter.

Agilent N5244A vector network analyzer was used to test the EMI shielding performance of PC/AgNW film in the frequency range of X‐band (8.2–12.4 GHz) and K‐band (18–26.5 GHz). The obtained scattering parameters (S_11_, S_12_, S_21_, and S_22_) were used to calculate the power coefficients and the EMI SE according to the following equations:^[^ ^48^ ^]^

(5) $$R=|S_{11}{|}^{2}=|S_{22}{|}^{2}$$

(6) $$T=|S_{12}{|}^{2}=|S_{21}{|}^{2}$$

(7) 1=A+R+T

(8) $$SE_{R}=-10\log\left(1-R\right)$$

(9) $$SE_{A}=-10\log\left(T/\left(1-R\right)\right)$$

(10) $$SE_{T}=SE_{R}+SE_{A}+SE_{M}$$

(11) $$\mathrm{Shielding}\;\mathrm{efficiency}\;\left(\%\right)\;=\;100-\left(1/10^{\left(SE/10\right)}\right)$$

Where A, R, and T are the coefficients of absorptivity, reflectivity, and transmission, respectively; SE_T_, SE_R_, SE_A_, and SE_M_ represent the total shielding effectiveness, microwave reflection, microwave absorption, and multiple reflections, respectively. For SE_A_ >10 dB, SE_M_ can usually be ignored.^[^ ^49^ ^]^ To evaluate the Joule heating performance of PC/AgNW film, a specific voltage was applied using the DC power (UNI‐T UTP1306S, Youlide Technology), and the surface temperature was recorded using an infrared thermal imaging instrument (FLIR‐E60).

## Conflict of Interest

The authors declare no conflict of interest.

## Supporting information

Supporting Information

## Data Availability

The data that support the findings of this study are available from the corresponding author upon reasonable request.
