# Supplementary material for: Centrifugal Inertia‐Induced Directional Alignment of AgNW Network for Preparing Transparent Electromagnetic Interference Shielding Films with Joule Heating Ability
Source: Adv Sci (Weinh). 2024 Aug 8;11(38):2406758. doi: 10.1002/advs.202406758 (PMC11481190; doi:10.1002/advs.202406758)
Supplement: Supplementary file 1 — Supporting Information [file ADVS-11-2406758-s001.docx]

**Supporting Information**

**Centrifugal Inertia-Induced Directional Alignment of AgNW Network for Preparing Transparent Electromagnetic Interference Shielding Films with Joule Heating Ability**

Weijun Zhao, Jingwen Dong, Zhaoyang Li, Bing Zhou*, Chuntai Liu*, Yuezhan Feng*

W. Zhao, J. Dong, Z. Li, B. Zhou, Prof. C. Liu, Dr. Y. Feng

State Key Laboratory of Structural Analysis, Optimization and CAE Software for Industrial Equipment,

National Engineering Research Center for Advanced Polymer Processing Technology, Zhengzhou University,

Zhengzhou 450002, China.

E-mail: zhoubing5115@zzu.edu.cn (B. Zhou), ctliu@zzu.edu.cn (C. Liu), yzfeng@zzu.edu.cn (Y. Feng)

**Figure S1**. Characterization of the synthesized AgNW. (a) SEM image. (b)AFM image. (c) TEM image. (d) UV-vis spectrum, the inset shows the Tyndall effect of AgNW aqueous solution.

AgNW with a tremendous aspect ratio was synthesized by polyol reduction method. SEM image shows that the synthesized AgNW has a uniform morphology and exhibits a typical one-dimensional linear structure with a length of about 25 μm (Figure S1a). In addition, the synthesized AgNW exhibits a mean diameter of approximately 80 nm, signifying an 1D threadlike nanostructure (Figure S1b). It is worth noting that the obtained AgNW is coated with a layer of PVP with a thickness of approximately 3 nm (Figure S1c). Moreover, the UV-vis absorption spectrum shows characteristic peaks of AgNW at 355nm and 392nm, indicating that the obtained AgNW has high purity. The Tyndall effect verifies that the synthesized AgNW has excellent dispersion stability in water.

**Figure S2.** Digital Picture of rotation spraying equipment.

**Figure S3.** The mass of an ideal single AgNW.

**Figure S4**. (a) FTIR spectra and (b) enlarged image of PC/AgNW film.

From the FTIR spectra shown in Figure S4, the characteristic peaks of PC film, and AgNW appeared in the PC/AgNW film, indicating that the conductive filler is successfully loaded onto the PC substrate. Notably, by local enlargement, the shift of the C=O band of PC/AgNW film (from 1770 cm^-1^ to 1768 cm^-1^) confirms the existence of a hydrogen bonding force between the conductive fillers and the substrate.

**Figure S5**. SEM of AgNW film with various AgNW coating density at 4000 rpm rotation speed.

**Figure S6**. (a) Optical transmittance and (b) sheet resistance and light transmittance (550 nm) of random AgNW network with various AgNW coating density.

**Figure S7**. (a) Temperature state (2V) of AgNW-428 film and (b) corresponding histogram of temperature statistics.

**Figure S8**. ΔR/R_0_ as a function of bending cycles (3 mm) (ΔR and R_0_ represent the resistance change and the initial resistance, respectively).

**Figure S9**. EMI shielding performance in K-band.

**Figure S10**. (a) Average EMI SE_R_, SE_A_, and SE_T_ values and (b) R-A coefficients of AgNW film with various AgNW coating density at the X-band. (c) Average EMI SE_R_, SE_A_, and SE_T_ values and (d) R-A coefficients of AgNW film at the K-band.

**Figure S11**. (a) Electrothermal conversion curves of AgNW-428 (0 rpm) film at different input voltages with random network. (b) The saturation temperature as a function of U^2^ of AgNW-428 (0 rpm) film. (c) Temperature state (4V) of AgNW-428 (0 rpm) film and (d) corresponding histogram of temperature statistics.

**Figure S12.** The home-made wireless power transmission system based on the Tesla coil, where the components corresponding to the Figure 5a are marked.

**Table S1.** Centrifugal force statistics at different speeds.

| **Speed/rpm** | 0 | 1000 | 2000 | 3000 | 4000 |
| --- | --- | --- | --- | --- | --- |
| **ω/rad*s^-1^** | 0 | 105 | 210 | 314 | 420 |
| **Centrifugal force (F)/N** | 0 | 0.65*10^-12^ | 2.62*10^-12^ | 5.86х10^-12^ | 10.48*10^-12^ |
| **f_p_** | 0.15 | 0.24 | 0.43 | 0.52 | 0.67 |

**Table S2.** Comparison of EMI shielding performances of various transparent conductive films.

| **Materials** | **Matrix** | **Transmittance**  **(%)** | **EMI SE**  **(dB)** | **Refs.** |
| --- | --- | --- | --- | --- |
| ITO | Quartz | 80 | 28 | ^[1]^ |
| PEI/RGO | PEI | 62  73 | 6.37  3.09 | ^[2]^ |
| graphene network fabric | PET | 70.85 | 12.86 | ^[3]^ |
| rGO/PMMA | PMMA | 80 | 10 | ^[4]^ |
| AgNW/PEDOT:PSS | Glass | 78.2 | 19.6 | ^[5]^ |
| MXene/AgNW-PVA | PVA | 52.3 | 32 | ^[6]^ |
| PC/MXene/Hf-SiO2 | PC | 50  62  78 | 10.5  7  3 | ^[7]^ |
| PDMS/AgNW | PDMS | 83.2  80.5  78.1 | 18.7  23.4  27.9 | ^[8]^ |
| AgNW@MXene/Wood | Transparent wood | 28.8 | 44 | ^[9]^ |
| PES/AgNW | PET | 90  85  81 | 5  16  25 | ^[10]^ |
| MXene/AgNW/TW | Wood | 31.7 | 31.5 | ^[11]^ |
| MXene/CNT | Glass | 41 | 3.5 | ^[12]^ |
| MXene/TPU | TPU | 43.1 | 10.9 | ^[13]^ |
| MXene/PET | PET | 45 | 10 | ^[14]^ |
| CU Mesh | Glass | 82 | 24 | ^[15]^ |
| A/RGO/SANW | PET | 85 | 24 | ^[16]^ |
| AgNW/PET mesh | PET | 67.8 | 44 | ^[17]^ |
| AgNW/PI | PI | 58 | 55 | ^[18]^ |
| This work | PC | 72.9 | 35.2 |  |

**References**

[1] Y. Ren, P. Liu, R. Liu, Y. Wang, Y. Wei, L. Jin; G. Zhao, *Jalloy. Compd.* **2022,** *893*, 162304.

[2] S. Kim, J.-S. Oh, M.-G. Kim, W. Jang, M. Wang, Y. Kim, H. W. Seo, Y. C. Kim, J.-H. Lee, Y. Lee; J.-D. Nam, *ACS Appl. Mater. Interfaces* **2014,** *6*, 17647.

[3] J. Han, X. Wang, Y. Qiu, J. Zhu; P. Hu, *Carbon* **2015,** *87*, 206.

[4] H.-L. Zhang, Y. Xia; J.-G. Gai, *ACS Omega* **2018,** *3*, 2765.

[5] X. Zhang, J. Shan, C. Liu, Z. Li, X. Guo, X. Zhao; H. Yang, *J. Materiomics* **2022,** *8*, 1191.

[6] B. Zhou, M. Su, D. Yang, G. Han, Y. Feng, B. Wang, J. Ma, J. Ma, C. Liu; C. Shen, *ACS Appl. Mater. Interfaces* **2020,** *12*, 40859.

[7] B. Zhou, Z. Li, Y. Li, X. Liu, J. Ma, Y. Feng, D. Zhang, C. He, C. Liu; C. Shen, *Compos. Sci. Technol.* **2021,** *201*, 108531.

[8] Y. Feng, J. Song, G. Han, B. Zhou, C. Liu; C. Shen, *Small Methods* **2023,** *7*, 2201490.

[9] M. Cheng, M. Ying, R. Zhao, L. Ji, H. Li, X. Liu, J. Zhang, Y. Li, X. Dong; X. Zhang, *ACS Nano* **2022,** *16*, 16996.

[10] M. Hu, J. Gao, Y. Dong, K. Li, G. Shan, S. Yang; R. K.-Y. Li, *Langmuir* **2012,** *28*, 7101.

[11] Z. Li, W. Che, Y. Jiang, Y. Liu, X. Fang; Y. Peng, *Colloid. Surfaces A* **2023,** *676*, 132211.

[12] G. M. Weng, J. Li, M. Alhabeb, C. Karpovich, H. Wang, J. Lipton, K. Maleski, J. Kong, E. Shaulsky, M. Elimelech, Y. Gogotsi; A. D. Taylor, *Adv. Funct. Mater.* **2018,** *28*, 1803360.

[13] Q. Li, Y. Sun, B. Zhou, G. Han, Y. Feng, C. Liu; C. Shen, *ACS Appl. Nano Mater.* **2023,** *6*, 3395.

[14] T. Yun, H. Kim, A. Iqbal, Y. S. Cho, G. S. Lee, M. K. Kim, S. J. Kim, D. Kim, Y. Gogotsi, S. O. Kim; C. M. Koo, *Adv. Mater.* **2020,** *32*, 1906769

[15] Y. Han, H. Zhong, N. Liu, Y. Liu, J. Lin; P. J. A. E. M. Jin, *Adv. Electron. Mater.* **2018,** *4*, 1800156.

[16] D. G. Kim, J. H. Choi, D.-K. Choi; S. W. Kim, *ACS Appl. Mater. Interfaces* **2018,** *10*, 29730.

[17] J. Gu, S. Hu, H. Ji, H. Feng, W. Zhao, J. Wei; M. J. N. Li, *Nanotechnology* **2020,** *31*, 185303.

[18] D.-H. Kim, Y. Kim; J.-W. Kim, *Mater. Design* **2016,** *89*, 703.
